# New Insight into Titanium–Magnesium Ziegler–Natta Catalysts Using Photoluminescence Spectroscopy

**DOI:** 10.1177/0003702820927434

**Published:** 2020-06-18

**Authors:** Valentina N. Panchenko, Anton I. Kostyukov, Anton Yu Shabalin, Evgeniy A. Paukshtis, Tatiana S. Glazneva, Sergei G. Kazarian

**Affiliations:** 1 Boreskov Institute of Catalysis, Novosibirsk, Russia; 2 Department of Natural Sciences, Novosibirsk State University, Novosibirsk, Russia; 3 Department of Chemical Engineering, Imperial College London, London, UK

**Keywords:** Titanium–magnesium catalysts, MgCl_2_, TiCl_4_, dibutyl phthalate, infrared spectroscopy, infrared, IR, photoluminescence spectroscopy

## Abstract

This paper presents the results of study of titanium–magnesium catalysts often used in polymerization processes, by photoluminescence spectroscopy (PL) in combination with diffuse reflectance infrared Fourier transform spectroscopy (DRIFTS). The interaction of dibutyl phthalate (DBP) with MgCl_2_ was studied at DBP_added_/Mg = 0–1 (mol/mol). The luminescence spectra with excitation at 278 nm and the excitation spectra for main emission bands were recorded. It was shown that DBP adsorbed on magnesium chloride, both in the form of donor–acceptor complexes (D^+^A^–^) and in the form of molecular complexes. At DBP_added_/Mg <0.15, the formation of D^+^A^–^ complexes occur predominantly; with an increase in DBP_added_/Mg, the fraction of molecular complexes increases. Molecular complexes are destroyed during the treatment of the support by TiCl_4_. In this case, the structure of magnesium chloride is disordered and new coordination–unsaturated sites are formed. This work is a first attempt to apply PL spectroscopy in combination with DRIFTS spectroscopy to study titanium–magnesium Ziegler–Natta catalysts. The application of PL spectroscopy to such systems made it possible to detect interactions within and between donor molecules, which would be particularly challenging to achieve using other spectroscopic methods. Both spectroscopic methods provided crucial information about the existence of two types of complexes on the sample surface which is important for tuning the synthesis procedure of the titanium–magnesium catalysts for olefin polymerization.

## Introduction

**Scheme 1.**
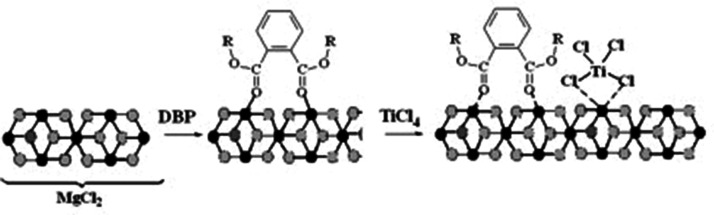
General scheme for the synthesis of TMC (black balls: Mg, light balls: Cl).

Titanium–magnesium catalysts (TMCs) for the stereospecific polymerization of ethylene have been known for over 60 years. TMCs are usually composed of magnesium chloride, electron donor base (phthalates, for example, dibutyl phthalate (DBP), malonates, alkoxysilanes, succinates, 1.3-diesters), and TiCl_4_ (Scheme 1).

Despite numerous studies, the formation of TMCs is still debatable, and this is primarily due to unresolved questions about the nature of the interaction of the Lewis bases (LB) with magnesium chloride and TiCl_4_. The specificity of the catalysts for olefin polymerization does not allow the contact of samples with air at any stages of their preparation, and the catalysts themselves operate at temperatures of 40–90 ℃. Therefore, exploring such systems is challenging.

Infrared (IR) spectroscopy and in particular diffuse reflectance infrared Fourier transform spectroscopy (DRIFTS) is one of the common physicochemical methods of analysis.^1–4^ It allows one to study the surface groups of a catalyst, evaluate acid–base properties, and study reaction mechanisms. IR spectroscopy is actively used to study TMCs. In the study of catalysts for olefin polymerization, the DRIFTS approach has an advantage over other modes of measurements in IR spectroscopy because it is very suitable for studies catalysts as powder and without contact of the catalysts with the atmosphere, i.e., in situ. Thus, the acid sites of magnesium chloride obtained from Mg and BuCl were studied using DRIFTS with CO as a probe molecule.^5^ Three bands were observed in the DRIFTS spectrum of CO adsorbed at room temperature: 2210, 2190, and 2170 cm^–1^, which the authors attributed to three-, four-, and five-coordinated Mg^2+^ ions. Later, low-temperature IR spectroscopy of CO adsorbed at MgCl_2_ obtained from the MgCl_2_ · 6C_2_H_5_OH adduct also revealed three bands at 2194, 2182, and 2163 cm^–1^.^6^ The authors attributed the band at 2182 cm^–1^ to CO complexes formed on four-coordinated magnesium ions of the (110) face, the band at 2163 cm^–1^ to five-coordinated magnesium ions of the (104) face, and the band at 2194 cm^–1^ to (012) and (015) faces containing five-coordinated magnesium ions. The higher ν(CO) value of CO complexes on five-coordinated magnesium ions of the (012) and (015) faces compared to CO complexes on four-coordinated magnesium ions of the (110) face was explained as the effect of chlorine ligands belonging to the neighboring layer on the CO molecule.

The interaction of DBP and ethyl benzoate (EB) with MgCl_2_ at LB/MgCl_2_ <0.3 (LB = DBP, EB) was studied by the IR spectroscopy.^7,8^ It was shown that esters are fixed on the surface of MgCl_2_ due to the interaction of the carbonyl group with the Lewis acid site (LAS) of magnesium chloride. In this case, surface complexes of the donor–acceptor type (D^+^A^–^) are formed. For malonates, alkoxysilanes, phthalates, succinates, 1,3-diesters, the existence of various forms of adsorption on the surface of magnesium chloride was shown by quantum chemical calculations. On the (104) face (sometimes referred to as (100)), both monodentate and bidentate (bridge) donor coordination are possible, and on the (110) face, bidentate (bridge), chelate, and interlayer (zip) donor coordination are possible.^9–11^ Depending on the O–O distance, a preferred form of donor adsorption on the (110) or (104) face is plausible.

It is worth noting that [MgCl_2_ · (LB)*_n_*]_x_ molecular complexes can be obtained from magnesium chloride, for example, the complexes with *n* = 2–3, where LB is HCOOC_2_H_5_, CH_3_COOC_2_H_5_, C_2_H_5_OH, dialkyl ketones.^12–15^ As was shown using X-ray diffraction (XRD), such adducts are characterized by a chain structure resembling the polymer [MgCl_2_ · nLB]_x_ chains.^12–15^ After chemical treatment, using TiCl_4_ or thermal activation, such structures are destroyed with the formation of δ-MgCl_2_, and the donor content becomes very low (*n* <0.3).^12,16^ Such molecular complexes can play a key role in the formation of the magnesium chloride, and thus studies investigating the structure of such [MgCl_2_ · (LB)*_n_*]_x_ adducts are of significant interest. Unfortunately, the molecular complexes were not detected using IR spectroscopy in TMC, they were observed using XRD in titanium adducts, e.g., [MgCl_2_ · (HCOOC_2_H_5_)*_k_* ·(TiCl_4_)*_m_*]_x_, 0.286 <*k* <0.5 and *m*/*k* ≈ 0.16.^12^ However, we do not exclude the presence of such adducts in ready-made TMCs. At the same time, IR spectroscopy does not allow the separation of molecular complexes and D^+^A^–^ complexes, since they have close absorption bands of stretching vibrations of carbonyl groups (ν_С=О_).

It is known that with γ irradiation of a magnesium chloride, F sites are formed that are characterized by 3.35–3.40 eV (400–394 nm) in the electronic spectrum, and by a signal of g = 2.0 with a half width of 50 ± 5 G in the EPR spectrum.^17^ Quantum chemical calculations show that in a well-ordered MgCl_2_ crystal, F sites are formed as a result of the removal of one Cl* atom, one or two Cl^–^ ions from (001) surface.^18^ In these works, it was suggested that such F sites can play an important role in the formation of TMCs. Furthermore, “activated” MgCl_2_ with a large number of defects on the surface is used for the synthesis of TMC.

Cheng et al.^19^ showed by DFT method the possibility of the existence of at least 12 defects on the (110) and (104) faces. These defects can contribute to the formation of F sites in MgCl_2_/LB samples and can possibly be identified by electron spectroscopy and photoluminescence (PL) spectroscopy.

Photoluminescence spectroscopy is one of the most sensitive research methods for determining low concentrations of luminescent compounds. The process of fluorescence and phosphorescence of organic compounds in solutions is usually described by the energy level diagram proposed by Kinno and Oblanka.^17^ Since nonradiative energy transfer depends on the electronic structure of the molecule and on the interaction of the molecule with the environment at short molecular distances, the PL spectra of organic molecules provide information on their interaction with neighboring molecules, which is usually not available for other spectral methods. In this case, phosphorescence spectra are especially informative. Phosphorescence is a slow process and so nonradiative processes of energy dissipation, for example, nonradiative deactivation, compete with it. Therefore, phosphorescence of organic molecules usually manifests itself in the gas phase, solution, or when the molecules are “rigidly” fixed in solution at low temperatures in an inert atmosphere, when interaction with the environment is minimized.^20,21^ In some cases, phosphorescence is observed without freezing, at room temperature. For example, 7-bromo-9,9-didodecylfluorene-2-carbaldehyde shows phosphorescence in a solution of CHCl_3_ and a polymethylmethacrylate (PMMA) matrix at 298 K in argon.^22^ It was shown that weakly photoluminescent 2,5-dihexyloxy-4-bromobenzaldehyde introduced into PMMA is capable of phosphorescing at 298 K.^23^

Phosphorescence is also characteristic of molecular complexes of the donor–acceptor D^+^A^–^ type. For example, there are complexes with charge transfer hexamethylbenzene–tetracyanbenzene; durene and mesitylene with phthalic or tetrachlorophthalic anhydrides; pyrene, perylene, and anthracene with amines.^20^ PL spectroscopy allows the study of the intermolecular interaction between adsorbed molecules on the surface. Nishikiori et al. managed to isolate the monomeric, dimeric, and larger associates of the 9-aminoacridine molecule on the surface of silica gel, since hydrogen bonds or other interactions lead to a bathochromic shift of the observed bands in the PL spectra.^24^

The study of TMCs is a challenging task, and this is related to the preparation of samples for analysis. These systems are unstable in the air, and minor changes in synthesis (temperature, solvent, DBP/Mg_added_ ratio, etc.) can extremely change the catalytic properties of the produced catalyst. Besides, real catalysts may differ greatly from the model systems. DRIFTS and PL spectroscopy allows exploring the real catalysts and controlling the process of their synthesis. These methods are available, simple to use, and allows identifying the reasons of uniqueness of a particular catalyst. Analysis of the literature showed that the PL spectroscopy was not previously used for the study of TMCs. However, the presence of coordination–unsaturated sites in magnesium chloride suggests the possibility of its use. We anticipated that this novel approach will allow us to establish fine details of the interaction of donor molecules with the surface of magnesium chloride.

The aim of this work was to identify various D^+^A^–^ complexes of the donor–acceptor type, as well as molecular complexes on the surface of magnesium chloride using PL spectroscopy in combination with IR spectroscopy. The formation of such complexes can be expected upon DBP adsorption as a result of the interaction of the oxygen of the carbonyl group of the donor with the LAS of the MgCl_2_ support. The results of this study provide a molecular-level insight for further developments in the synthesis of titanium–magnesium Ziegler–Natta catalysts.

## Experimental

All of the chemical reagents and solvents used in this study, heptane, chlorobenzene (PhCl) and DBP, buthyl chloride (BuCl), were dried over molecular sieves.

### Synthesis of MgCl_2_(BuCl)

An activated MgCl_2_ sample was synthesized accordingly to described procedure by reaction of magnesium with BuCl (at a molar ratio BuCl/Mg = 3) in n-heptane at 98 ℃, and then washed twice with the same solvent.^25^ MgCl_2_(BuCl) sample (S_BET_ = 70 m^2^/g) contained ∼10 wt.% of organic products.

### Synthesis of MgCl_2_/nDBP

As a standard procedure, a suspension of MgCl_2_(BuCl) in PhCl containing 1 g MgCl_2_/L was prepared in a reactor purged with dry argon by addition of PhCl to the MgCl_2_ powder under vigorous stirring. The suspension was then treated with a solution of donor (DBP) in PhCl (C_donor_ = 0.2 M), at a molar ratio of donor/MgCl_2_ = 0.1 or 1 at 115℃ for 1 h under a continuous flow of argon. After this reaction, chlorobenzene was decanted and the sample was washed twice with hot (115℃) PhCl and three times with heptane at room temperature, in portions of 20 mL per 1 g of MgCl_2_.

### Synthesis of MgCl_2_/DBP/TiCl_4_

As a standard procedure, a suspension of MgCl_2_/D in PhCl containing 40 g MgCl_2_/L was prepared in a reactor purged with dry argon by addition of PhCl to the MgCl_2_ powder under vigorous stirring. The suspension was then treated with a solution of TiCl_4_ in PhCl, at a molar ratio of Mg/Ti = 100 at 115℃ for 1 h under a continuous flow of argon. After this reaction, chlorobenzene was decanted and the sample was washed twice with hot (115℃) PhCl and three times with heptane at room temperature, in portions of 20 mL per gram of MgCl_2_.

### Chemical analysis

The Mg and Ti contents of the samples were determined using inductively coupled plasma -- atomic emission spectroscopy (ICP-AES) on an Optima 4300 DV (PerkinElmer) spectrometer, while the contents of DBP were determined by high-performance liquid chromatography in isocratic mode by using standard solutions of the compounds in acetonitrile. The measurements were made on an LC-20 Prominence (Shimadzu) liquid chromatograph.

### Diffuse Reflectance Infrared Fourier Transform Spectroscopy

All DRIFTS spectra were acquired on samples under an inert Ar atmosphere. All samples were dried under vacuum to a residual pressure of 2 × 10^–2^ mbar before measurements. Samples (0.2–0.3 g) were transferred, under an inert atmosphere, to a cell suitable for DRIFTS measurements. DRIFTS spectra were acquired with an FTIR-8400S (Shimadzu) spectrometer equipped with deuterated lanthanum α alanine-doped triglycine sulfate detector and a DRS-8000 attachment in the range of 400–6000 cm^–1^ with a resolution of 4 cm^–1^. All spectra are presented in F(R) Kubelka–Munk scale: F(R) = (1 − R)^2^/2R, where R is the reflection coefficient.

### Photoluminescence Measurements

PL spectra (λ_excitation_ = 278 nm) and photoluminescence excitation (PLE) spectra of the samples were obtained using an Eclipse (Cary) spectrofluorimeter.

## Results and Discussion

### MgCl_2_/nDBP Samples Study Using DRIFTS

For the study, MgCl_2_/nDBP samples were prepared by applying DBP on magnesium chloride at DBP_added_/MgCl_2_ (mol/mol) equal to 0.1, 0.15, 0.4, and 1. Chemical analysis data are shown in Table I.

**Table I.** Chemical analysis data of MgCl_2_/DBP supports and corresponding MgCl_2_/DBP/TiCl_4_ catalysts.

Entry	DBP_add_/Mg	MgCl_2_/DBP		MgCl_2_/DBP/TiCl_4_			
		DBP_ads_ (wt%)	DBP_ads_/Mg (mol/mol)	DBP_ads_ (wt%)	DBP_ads_/Mg (mol/mol)	ΔDBP (µmol/g)	Ti (wt%) (µmol/g)
1	0.10	6.2	0.03	–	–	–	–
2	0.15	8.3	0.04	4.2	0.02	148	0.9 (188)
3	0.40	28.8	0.15	13.2	0.06	561	0.9 (188)
4	1.0	34.5	0.20	–	–	–	–

It can be seen that the amount of adsorbed donor in MgCl_2_/nDBP samples increases with an increase in DBP_added_/MgCl_2_. The amount of adsorbed donor (DBP_ads_) is less than the introduced DBP and is 20–30% of DBP_added_ in all samples. This is indicated by a lower DBP_ads_/Mg value in the samples. A similar result was obtained for TMC prepared from Mg(OEt)_2_, TiCl_4_, and DBP at DBP_addeed_/Mg = 0.15.^26^ Such TMC had a molar ratio of DBP_ads_/Mg equal to 0.067 and the content of DBP of 13.5 wt%.

**Figure 1.**
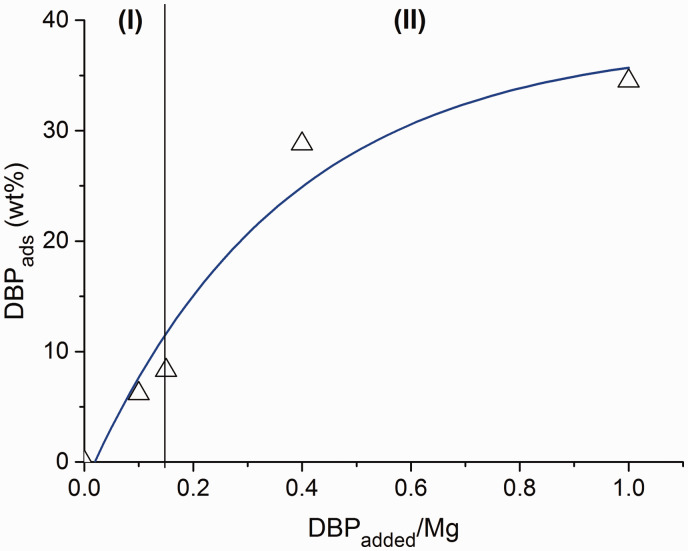
Dependence of the amount of fixed DBP on the molar ratio of introduced DBP to magnesium for MgCl_2_(BuCl)/nDBP samples.

Figure 1 shows the effect of the DBP_added_/Mg molar ratio on the amount of fixed DBP (DBP_ads_). It can be seen that as the DBP_added_/Mg molar ratio increased, there was an increase in the donor content in the samples. Moreover, in the samples obtained at DBP_added_/Mg <0.15 (region I), the adsorbed DBP_ads_ increases linearly, and in the samples obtained at DBP_added_/Mg >0.15 (region II), it changes drastically. The data suggest that the interaction of DBP in these regions with magnesium chloride occurs in different ways. It is possible that in the region I, the donor is adsorbed onto the LASs of the MgCl_2_ support with the formation of D^+^A^–^ donor–acceptor complexes, and in the region II, the formation of molecular complexes takes place.

**Figure 2.**
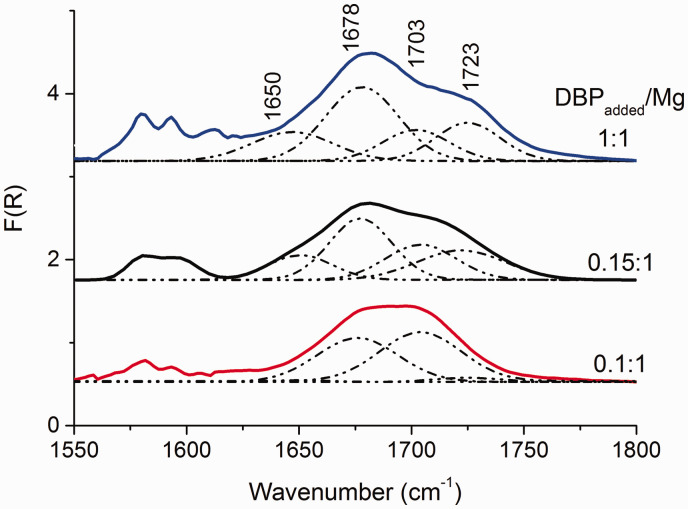
DRIFTS spectra of MgCl_2_(BuCl)/nDBP samples obtained at various DBP_added_/Mg ratios.

Figure 2 shows the DRIFTS spectra acquired from MgCl_2_/nDBP samples in the vibrational region of DBP carbonyl groups. It can be seen that the shape of the spectra of the samples at all molar ratios of DBP_added_/Mg are similar.

It is known that the spectral bands of the IR spectrum of MgCl_2_/nLB supports is a superposition of a large number of components characterizing the state of an adsorbed donor.^27^ The deconvolution of the MgCl_2_/nDBP spectra (Fig. 2) into four components was made based on the assumption of DBP adsorption on three types of LAS and physical adsorption. It is a simplified model that allows, as a first approximation, one to estimate the surface state of an adsorbed donor. As a result of the deconvolution of the spectra into four components, the bands at 1648, 1672, 1702, and 1723 cm^–1^, characterizing the stretching vibrations (ν_С=О_) of adsorbed phthalate, were revealed.

The bands attributed to DBP adsorbed onto Mg ions are shown in 1640–1670 cm^–1^ region from three-coordinated Mg ions, 1670–1690 cm^–1^ from four-coordinated Mg ions of the (110) face, 1690–1710 cm^–1^ from five-coordinated Mg ions of the (104) face, and the bands above 1710 cm^–1^ were weakly bound carbonyl groups.^28^

Furthermore, three-coordinated Mg ions are stronger LAS than sites formed on four- and five-coordinated magnesium ions; therefore, the bands of molecules adsorbed on these sites should appear in the DRIFTS spectra first.^5,29^ However, the band at 1650 cm^–1^ appears later, and its intensity increases with increase in DBP concentration. This suggests that 1650 cm^–1^ characterizes the adsorption of DBP on other LAS, for example, on four-coordinated magnesium ions.

The formation of [MgCl_2_ · (LB)_x_]_n_ molecular complexes during the synthesis of the MgCl_2_/LB support has been suggested previously.^30,31^ Polymeric [MgCl_2_ · (LB)_x_]_n_ complexes were synthesized and characterized in many works. For example, MgCl_2_ · (LB)_2_, where LB is ethyl acetate, ethyl propionate, EB, ethyl crotonate, acetone or 2-butanone, ethanol, THF, [Mg_2_Cl_4_(1,3-dimethoxypropane)_2_(H_2_O)].^12,32–34^

The DFT calculations also show the possibility of the formation of molecular complexes upon adsorption of donors (1,3-diethers, alkoxysilanes, and succinates) on magnesium chloride.^35^ In such complexes, two oxygen atoms of one or two LB are bound to a four-coordinated magnesium ion.

Since donor adsorption is insular in nature, we can hypothesize the formation of similar complexes with DBP in MgCl_2_/nDBP samples. However, for a site containing two adsorbed LB molecules, two absorption bands should be observed in the DRIFTS spectrum (ν_s_ and ν_as_).

Probably, after adsorption of the second DBP molecule, the cleavage of the band at 1678 cm^–1^ into two bands should occur, with one of the bands being at 1650 cm^–1^, and the second band in the region above 1780 cm^–1^. However, the spectral bands in the 1730–1800 cm^–1^ region are complex, so it is not yet possible to isolate it.

The DFT calculations show that bidentate donors, DEP, DIBP, *p*-iso-propoxy ethyl benzoate, *p*-ethoxy ethyl benzoate, and *p-tert*-butyl ethyl benzoate may have interlayer (zip) coordination on the (110) MgCl_2_ face.^11^ All this suggests the formation of molecular complexes in MgCl_2_/nDBP samples obtained with DBP_added_/Mg >0. With high donor content, such adsorption can lead to the destruction of the structure of magnesium chloride. Thus, it can be assumed that in region I (Fig. 1), DBP adsorption occurs on the LAS with the formation of ionic complexes, and molecular complexes begin to form in region II.

### MgCl_2_/nDBP Samples Study by Photoluminescence

**Figure 3.**
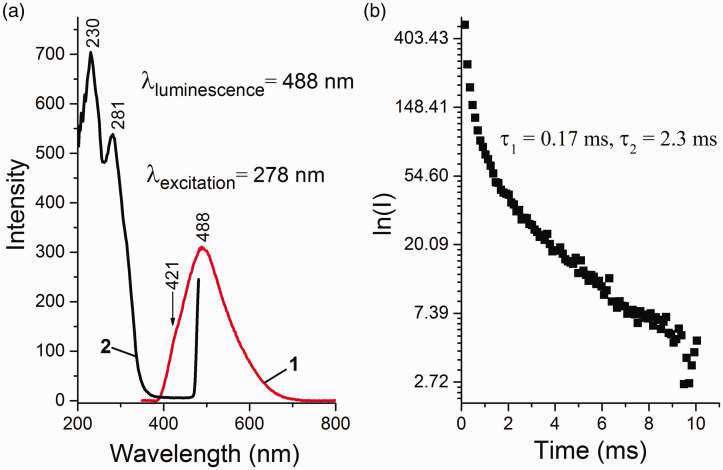
(a) PL (1) and PLE (2) spectra and (b) decay kinetics of MgCl_2_.

The source MgCl_2_ was studied by photoluminescent spectroscopy. Analysis of the literature data showed that λ_excitation_ = 278 nm can be used to measure the PL spectra of DBP.^20,22^ The PL spectrum of the MgCl_2_ sample obtained at λ_excitation_ = 278 nm is shown in Fig. 3. The PL spectrum contains a wide band with a maximum at 488 nm and a shoulder at 421 nm. The obtained PLE spectrum for λ_max_ = 488 nm is characterized by strong bands in the ultraviolet spectral region with maxima at 230 and 281 nm. The nature of this PL is most likely related to possible organic impurities, as well as the possible manifestation of anionic defects in the form of Cl^−^ vacancies.^18^ The kinetics of PL decay with λ_max_ = 488 nm is described by a biexponential dependence and has lifetimes of 0.17 and 2.3 ms typical for phosphorescence (τ_1_ and τ_2_).

The PL spectrum of the DBP heptane solution contains the bands 371, 414, 436, and 470 nm (Fig. S1, Supplemental Material). Since luminescence decay occurs at a high rate, we can conclude that they characterize S_1_* → S_0_, i.e., only fluorescence is observed in a DBP solution at room temperature.

**Figure 4.**
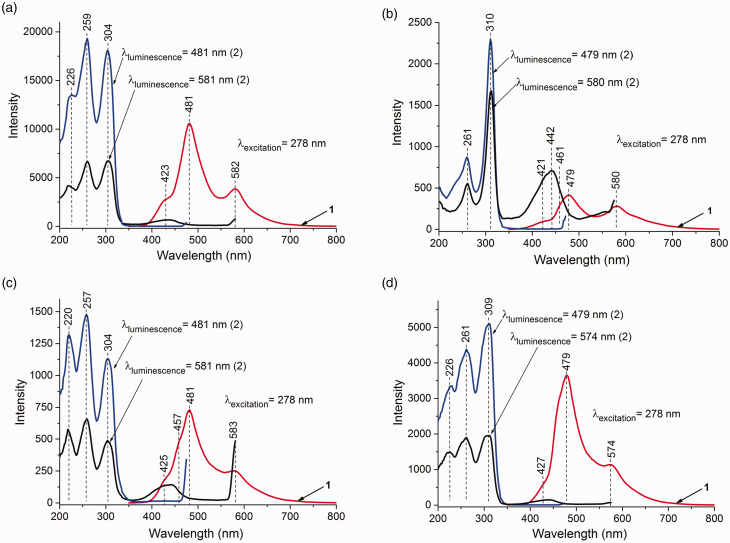
PL (1) and PLE (2) spectra of samples at λ_excitation_ = 278 nm: (a) MgCl_2_/0.15DBP, (b) MgCl_2_/0.4DBP, (c) MgCl_2_/0.15DBP/TiCl_4_, and (d) MgCl_2_/0.4DBP/TiCl_4_.

**Figure 5.**
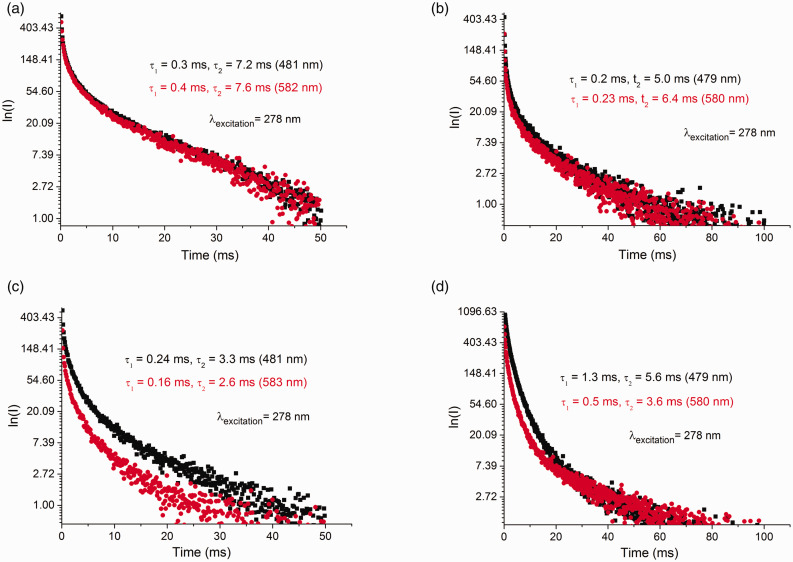
Decay kinetics for samples at λ_excitation_ = 278 nm: (a) MgCl_2_/0.15DBP, (b) MgCl_2_/0.4DBP, (c) MgCl_2_/0.15DBP/TiCl_4_, and (d) MgCl_2_/0.4DBP/TiCl_4_.

The PL spectra of MgCl_2_/nDBP samples with *n* = 0.1, 0.15, 0.4, and 1.0 are shown in Fig. 4 and Fig. S2. The spectra of all samples contain two bands with maxima near 479–482 and 579–583 nm. The decay time (τ) of these bands is shown in Fig. 5. The decay time is in the millisecond interval that is characteristic of phosphorescence. The values of τ for the samples are close to each other and significantly differ from τ for MgCl_2_. This indicates that the PL sites with maxima of 479–482, 579–583, and 620 nm are of a different nature from that of MgCl_2_ and characterize adsorbed DBP. A decrease of τ with a rise in DBP_added_/Mg indicates an increase in the concentration of “quenching” atoms, i.e., the amount of DBP adsorbed on the support surface. The decay kinetics of all the above bands are described by their biexponential dependence. τ depends on many parameters: on energy relaxation processes and on external conditions (e.g., temperature, pressure, concentration of luminescent atoms and molecules, concentration of “quenching” atoms and molecules, etc.). It can be assumed that the biexponential dependence τ is associated with the existence of various defects on the surface of magnesium chloride that act as electron “traps” upon excitation.

In the PLE spectra of the samples, bands similar in structure with maxima at 259 (261) and 301 (310) nm, characterizing DBP, are observed. The PLE spectra of the samples were similar in their positions to the DR ultraviolet–visible spectra (Fig. S3). The band at 259 (261) nm characterizes π–π transitions of the aromatic ring, and 301 (310) nm characterizes n–π transitions of the carbonyl group. Higher values of the band characterizing the n–π transitions in MgCl_2_/nDBP samples compared to the DBP solution (290 nm) may indicate the interaction of DBP carbonyl groups with the LAS of the support with the formation of D^+^A^–^ donor–acceptor surface complexes.^36^ An increase in the donor content in the samples resulted in a bathochromic shift of the band in the PLE spectrum which characterized the n–π transition of the carbonyl group. This indicated a weakening of the bond between the carbonyl group of the donor and the LAS support. The intensity ratio I_π–π_/ I_n–π_ decreases from 1.3 to 0.2. This shows that the number of D^+^A^–^ ion pairs is reduced. This was probably due to a change in the structure of magnesium chloride and the formation of polymer molecular complexes.

Thus, analysis of the PL and PLE spectra of MgCl_2_/nDBP samples indicated that the bands at 479–482 and 579–583 nm are similar in nature and characterized ionic complexes of adsorbed DBP with the transition energy of T_1_* → S_0_ equal to 2.82 and 2.34 eV, respectively. Surface D^+^A^–^ complexes characterized by a band at 479–482 nm are stronger than the complexes characterized by a band at 579–583 nm.

The presence of two bands in the PL spectra of MgCl_2_/nDBP samples indicated the formation of two different D^+^A^–^ complexes of DBP. Since there are four and five coordinatively unsaturated magnesium ions on the surface of magnesium chloride, it could be assumed that the appearance of two bands in the PL spectrum is associated with this. On the other hand, the long-wavelength shift of the bands in the PL spectra of these samples can be associated with interactions with neighboring molecules, for example, chlorine atoms or OH groups, always present in magnesium chloride due to the trace amounts of water.^25,37^

### Study of the Interaction of MgCl_2_/nDBP with TiCl_4_

It has been shown that the polymer [MgCl_2_ · (LB)_x_]_n_ molecular complexes with *n* = 2, 3 are destroyed by interaction with TiCl_4_ or thermal activation with the formation of δ–MgCl_2_.^13,38^ Therefore, for a more accurate identification of molecular complexes, the interaction of MgCl_2_/0.15DBP samples (region I) and MgCl_2_/0.4DBP (region II) with a TiCl_4_ solution at Ti/Mg = 100 was studied.

The chemical analysis is summarized in Table I. The application of TiCl_4_ leads to a decrease in the donor content by 49.50–54.15%. The number of magnesium atoms in these samples per one DBP molecule increases from 25 to 50 and from 6.6 to 17, respectively. The TiCl_4_ content in the samples is small (0.9 wt.%), and it differs from the amount of desorbed DBP. In the MgCl_2_/0.15DBP/TiCl_4_ sample, the amount of adsorbed titanium tetrachloride is 30% more than the amount of removed donor, while in the MgCl_2_/0.4DBP/TiCl_4_ sample, the amount of adsorbed TiCl_4_ is three times higher than that of desorbed DBP.

**Figure 6.**
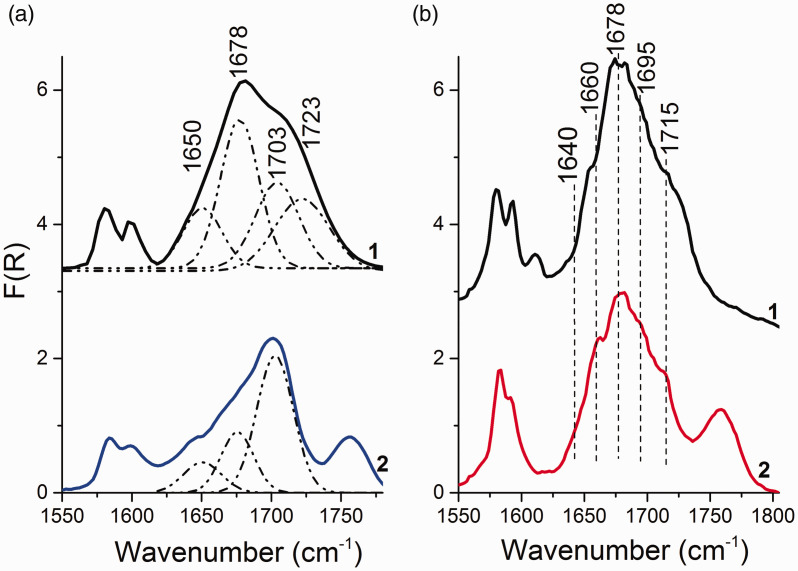
DRIFTS spectra of (1) MgCl_2_/nDBP supports and (2) corresponding MgCl_2_/nDBP/TiCl_4_ catalysts: (a) *n* = 0.15 and (b) *n* = 0.4.

Figure 6 shows the DRIFTS spectra of MgCl_2_/0.15DBP and MgCl_2_/0.4DBP samples before and after treatment with a TiCl_4_ solution. The deposition of TiCl_4_ on the supports leads to the spectrum shape change. In the DRIFTS spectrum of the MgCl_2_/0.15DBP/TiCl_4_ sample, the relative intensity of bands at 1650 and 1678 cm^–1^ decreases and the band at 1723 cm^–1^ disappears (Fig. 6a). In the spectra of the MgCl_2_/0.4DBP/TiCl_4_ sample, a decrease in the relative intensity of bands at 1650, 1678, and 1723 cm^–1^ is observed (Fig. 6b). In addition, new bands at 1640, 1660, and 1715 cm^–1^ appear. A sharp decrease in the relative intensity of bands at 1678 and 1723 cm^–1^ indicates the removal of DBP adsorbed on four-coordinated Mg ions.

Figure 4 shows the PL spectra of the studied catalysts. Two bands with maxima at 480–482 and 580–582 nm can also be distinguished in the spectra. The PL spectra of the different samples were very similar.

It is noteworthy that the decrease in DBP concentration (Fig. 4) in the MgCl_2_/0.15DBP/TiCl_4_ sample leads t a decrease in PL band intensity (12 000 → 750 for λ_481_) and to an increase in that for MgCl_2_/0.4DBP sample (400 → 3800 for λ_481_). This shows that the number of D^+^A^–^ ion complexes in the MgCl_2_/0.15DBP/TiCl_4_ sample decreases. The number of D^+^A^–^ ion complexes was found to increase for the MgCl_2_/0.4DBP/TiCl_4_ sample, probably as a result of an increase in the number of defects in the MgCl_2_ support.

Numerous XRD studies of [MgCl_2_ · (LB)_x_]_n_ molecular complexes with *n* = 2–3 containing monodentate donors (e.g., COOC_2_H_5_, CH_3_COOC_2_H_5_, C_2_H_5_OH) show that such complexes have well-ordered crystalline structure with donors in axial position.^12,14,15^ However, chemical or temperature activation removes most of LB, which leads to the destruction of the complexes structure. In this case, δ-MgCl_2_ is formed. Our data show that the treatment of the MgCl_2_/0.4DBP support with a TiCl_4_ solution results in increase in the number of LAS capable of adsorbing DBP with the formation of D^+^A^–^ ion complexes and this was probably due to the destruction of molecular complexes.

The phosphorescence lifetime τ of the observed bands at 482 and 582 nm also changes when TiCl_4_ is deposited on MgCl_2_/0.15DBP and MgCl_2_/1.0DBP supports. The values of τ_1_ and τ_2_ decrease. The reason for this may be an increase in the rate of nonradiative conversion of the absorbed radiation energy and the rate of quenching of the triplet state by “impurities” which are adsorbed TiCl_4_ molecules. This suggested that the adsorbed TiCl_4_ molecule was located in the first coordination sphere of DBP, i.e., it is a part of the precursor of the active site.

## Conclusion

The interaction of DBP with MgCl_2_ was studied by PL spectroscopy in combination with DRIFTS. DRIFTS showed how the DBP interacted with the LAS of the magnesium chloride support. PL spectroscopy revealed the formation of both D^+^A^–^ donor–acceptor complexes and molecular complexes on the surface of magnesium chloride. Part of the ionic D^+^A^–^ complexes probably interacted with neighboring surface Cl^−^ or OH^−^ groups. Their amount depended on the DBP_added_/Mg molar ratio used for the synthesis of the support. Molecular complexes began to form in MgCl_2_/nDBP samples at DBP_added_/Mg >0.1.

Photoluminescence spectroscopy revealed that treatment of MgCl_2_/nDBP supports with a TiCl_4_ not only led to the removal of the donor from the surface of magnesium chloride and to the formation of new LAS capable of adsorbing titanium or an organoaluminum activator, but also resulted in the destruction of molecular complexes. This method allowed us to explain why the synthesis of the TMC for olefin polymerization is preferable to perform at a molar ratio of DBP/Mg_added_ below 0.2.

## Supplemental Material

sj-pdf-1-asp-10.1177_0003702820927434 - Supplemental material for New Insight into Titanium–Magnesium Ziegler–Natta Catalysts Using Photoluminescence Spectroscopy Click here for additional data file. Supplemental material, sj-pdf-1-asp-10.1177_0003702820927434 for New Insight into Titanium–Magnesium Ziegler–Natta Catalysts Using Photoluminescence Spectroscopy by Valentina N. Panchenko, Anton I. Kostyukov, Anton Yu Shabalin, Evgeniy A. Paukshtis, Tatiana S. Glazneva and Sergei G. Kazarian in Applied Spectroscopy

## Supplemental Material

The supplemental material mentioned in the text, consisting of Figs. S1 to S3, is available in the online version of the journal.

## References

[1] Lamberti C. Zecchina A. Groppo E., et al. “Probing the Surfaces of Heterogeneous Catalysts by in Situ IR Spectroscopy”. Chem. Soc. Rev. 2010, 39(12): 4951–5001. 21038053 10.1039/c0cs00117a

[2] Bache Ø. Ystenes M. “Double-Chamber Flow Cell for in Situ Infrared Spectroscopy Studies of Chemical Reactions in Ziegler–Natta Catalyst Systems”. Appl. Spectrosc. 1994, 48(8): 985–993.

[3] Pezzolo A.L. Colombi M. Mazzocchin G.A. “Spectroscopic and Chemometric Comparison of Local River Sands with the Aggregate Component in Mortars from Ancient Roman Buildings Located in the X Regio Between the Livenza and Tagliamento Rivers, Northeast Italy”. Appl. Spectrosc. 2018, 72(10): 1528–1537. 29972314 10.1177/0003702818789140

[4] Gomora-Herrera D. Bolanos J.N. Lijanova I.V., et al. “Study of Surface Wettability Change of Unconsolidated Sand Using Diffuse Reflectance Infrared Fourier Transform Spectroscopy and Thermogravimetric Analysis”. Appl. Spectrosc. 2018, 72(4): 562–572. 29218999 10.1177/0003702817750640

[5] Paukshtis E.A. Zakharov V.A. Makhtarulin S.I., et al. “A Study of Surface Acid Sites in Highly Dispersed Magnesium–Chloride and Titanium Magnesium Catalysts for Ethylene Polymerization by Diffuse-Reflectance IR Spectroscopy”. Kinet. Catal. 1994, 35(6): 848–852.

[6] D’Amore M. Thushara K.S. Piovano A., et al. “Surface Investigation and Morphological Analysis of Structurally Disordered MgCl_2_ and MgCl_2_/TiCl_4_ Ziegler–Natta Catalysts”. ACS Catal. 2016, 6(9): 5786–5796.

[7] Song W.-D. Chu K.-J. Chang H.-S., et al. “Model for the Formation of Active Titanium Complexes on MgCl_2_-Ethyl Benzoate-TiCl_4_ Catalysts for Propylene Polymerization”. J. Mol. Catal. 1993, 84(1): L109–L116.

[8] Potapov A.G. Bukatov G.D. Zakharov V.A. “DRIFTS Study of Internal Donors in Supported Ziegler–Natta Catalysts”. J. Mol. Catal. A: Chem. 2006, 246(1–2): 248–254.

[9] Correa A. Piemontesi F. Morini G., et al. “Key Elements in the Structure and Function Relationship of the MgCl_2_/TiCl_4_/Lewis Base Ziegler–Natta Catalytic System”. Macromolecules. 2007, 40(25): 9181–9189.

[10] Singh G. Kaur S. Makwana U., et al. “Inﬂuence of Internal Donors on the Performance and Structure of MgCl_2_ Supported Titanium Catalysts for Propylene Polymerization”. Macromol. Chem. Phys. 2009, 210(1): 69–76.

[11] Vanka K. Singh G. Iyer D., et al. “DFT Study of Lewis Base Interactions with the MgCl_2_ Surface in the Ziegler–Natta Catalytic System: Expanding the Role of the Donors”. J. Phys. Chem. C. 2010, 114(37): 15771–15781.

[12] Auriemma F. De Rosa C. “Formation of (MgCl_2_)X Polynuclear Species During Preparation of Active MgCl_2_ Supported Ziegler–Natta Catalysts from Solid Solvates with Lewis Bases”. Chem. Mater. 2007, 19(24): 5803–5805.

[13] Auriemma F. De Rosa C. “Theoretical Investigation of (MgCl_2_)X Polynuclear Species Formed During Preparation of MgCl_2_-Supported Ziegler–Natta Catalysts from Solid Solvates”. J. Appl. Crystallogr. 2008, 41(1): 68–82.

[14] Thushara K.S. Gnanakumar E.S. Mathew R. Ajithkumar T.G., et al. “MgCl_2_ · 4((CH_3_)_2_CHCH_2_OH): A New Molecular Adduct for the Preparation of TiCl*_x_*/MgCl_2_ Catalyst for Olefin Polymerization”. Dalton Trans. 2012, 41(37): 11311–11318. 22886043 10.1039/c2dt31211e

[15] Di Noto V. Zannetti R. Viviani M., et al. “MgCl_2_-Supported Ziegler–Natta Catalysts: A Structural Investigation by X-ray Diffraction and Fourier Transform IR Spectroscopy on the Chemical Activation Process Through MgCl_2_-Ethanol Adducts”. Makromol. Chem. 1992, 193(7): 1653–1663.

[16] Di Noto V. Bresadola S. “New Synthesis of a Highly Active δ-MgCl_2_ for MgCl_2_/TiCl_4_/AlEt_3_ Catalytic Systems”. Macromol. Chem. Phys. 1996, 197(11): 3827–3835.

[17] Kinno S. Onaka R. “ESR and Optical Studies on Γ-Irradiated MgCl_2_ Single Crystals”. J. Phys. Soc. Japan. 1983, 52(1): 267–271.

[18] Costuas K. Parrinello M. “Structure and Chemical Activity of Point Defects on MgCl_2_ (001) Surface”. J. Phys. Chem. B. 2002, 106(17): 4477–4481.

[19] Cheng R. Luo J. Liu Z., et al. “Adsorption of TiCl_4_ and Electron Donor on Defective MgCl_2_ Surfaces and Propylene Polymerization over Ziegler–Natta Catalyst: A DFT Study”. Chin. J. Polym. Sci. 2013, 31(4): 591–600.

[20] McGlynn S.P. Azumi T. Kinoshita M. Molecular Spectroscopy of the Triplet State., Englewood Cliffs, NJ: Prentice-Hall, Inc., 1969. P. 448.

[21] Purdy B.B. Hurtubise R.J. “Changes in the Photophysical Properties with Heavy Atoms and the Effects of Modulus for 4-Phenylphenol in Solid-Matrix Luminescence”. Appl. Spectrosc. 1992, 46(6): 988–993.

[22] Xu J. Takai A. Kobayashi Y., et al. “Phosphorescence from a Pure Organic Fluorene Derivative in Solution at Room Temperature”. Chem. Commun. 2013, 49(76): 8447–8449. 10.1039/c3cc44809f 23939484

[23] Lee D. Bolton O. Kim B.C., et al. “Room Temperature Phosphorescence of Metal-Free Organic Materials in Amorphous Polymer Matrices”. J. Am. Chem. Soc. 2013, 135(16): 6325–6329. 23521108 10.1021/ja401769g

[24] Nishikiori H. Tanaka N. Minami Y., et al. “Molecular Forms and Fluorescence Processes of 9-Aminoacridine in Thin Sol–Gel Films”. J. Photochem. Photobiol., A. 2010, 212(1): 62–67.

[25] Potapov A.G. Bukatov G.D. Zakharov V.A. “DRIFTSS Study of the Interaction of the AlEt_3_ Cocatalyst with the Internal Donor Ethyl Benzoate in Supported Ziegler–Natta Catalysts”. J. Mol. Catal. A: Chem. 2009, 301(1–2): 18–23.

[26] Chumachenko N.N. Zakharov V.A. Bukatov G.D., et al. “A Study of the Formation Process of Titanium–Magnesium Catalyst for Propylene Polymerization”. Appl. Catal. A. 2014, 469: 512–516.

[27] Vanka K. Singh G. Iyer D., et al. “DFT Study of Lewis Base Interactions with the MgCl_2_ Surface in the Ziegler–Natta Catalytic System: Expanding the Role of the Donors”. J. Phys. Chem. C. 2010, 114(37): 15771–15781.

[28] Cheruvathur A.V. Langner E.H.G. Niemantsverdriet J.W., et al. “In Situ ATR-FTIR Studies on MgCl_2_-Diisobutyl Phthalate Interactions in Thin Film Ziegler–Natta Catalysts”. Langmuir. 2012, 28(5): 2643–2651. 22216939 10.1021/la203972k

[29] Trubitsyn D.A. Zakharov V.A. Zakharov I.I. “A Theoretical Investigation of the Adsorption Surface Sites of the Activated MgCl_2_”. J. Mol. Catal. A: Chem. 2007, 270(1–2): 164–170.

[30] Keszler B. Grobler A. Takàcs E., et al. “Studies on Highly Active Coordination Catalysts for Polymerization of α-Olefins: 2. Thermal Investigations of the Support System Anhydrous Magnesium Chloride–Ethyl Benzoate”. Polymer. 1981, 22(6): 818–821.

[31] Stukalov D.V. Zakharov V.A. Potapov A.G., et al. “Supported Ziegler–Natta Catalysts for Propylene Polymerization. Study of Surface Species Formed at Interaction of Electron Donors and TiCl_4_ with Activated MgCl_2_”. J. Catal. 2009, 266(1): 39–49.

[32] Di Noto V. Zanetti R. Bresadola S., et al. “Synthesis and Crystal Structure of the MgCl_2_ (CH_3_COOC_2_H_5_)_2_ · 12(CH_3_COOC_2_H_5_)”. Inorg. Chim. Acta. 1991, 190(2): 279–283.

[33] Di Noto V. Bandoli G. Dolmella A., et al. “Crystal Structure of Two Cocrystallized Complexes Obtained from the Reaction of Magnesium Chloride with 2,4-Pentanedione”. J. Chem. Crystallogr. 1995, 25(7): 375–378.

[34] Nissinen V.H. Koshevoy I.O. Pakkanen T.T. “Crystalline Magnesium Chloride–Electron Donor Complexes: New Support Materials for Ziegler–Natta Catalysts”. Dalton Trans. 2017, 46(13): 4452–4458. 28304034 10.1039/c7dt00193b

[35] Correa A. Credendino R. Pater J.T.M., et al. “Theoretical Investigation of Active Sites at the Corners of MgCl_2_ Crystallites in Supported Ziegler − Natta Catalysts”. Macromolecules. 2012, 45(9): 3695–3701.

[36] NIST Chemistry Webbook. Dibutyl phthalate. https://webbook.nist.gov/cgi/cbook.cgi?ID=84-74-2 [accessed Mar 20 2020].

[37] Arzoumanidis G.G. Karayannis N.M. “Infrared Spectral Characterization of Supported Propene Polymerization Catalysts: A Link to Catalyst Performance”. Appl. Catal. 1991, 76(2): 221–231.

[38] Di Noto V. Bresadola S. “New Synthesis of a Highly Active δ-MgC_12_ for MgC_12_/TiCl_4_/Alet3 Catalytic Systems”. Macromol. Chem. Phys. 1996, 197(11): 3827–3835.

